# Antibacterial activities of the methanol extracts of ten Cameroonian vegetables against Gram-negative multidrug-resistant bacteria

**DOI:** 10.1186/1472-6882-13-26

**Published:** 2013-01-31

**Authors:** Jaurès AK Noumedem, Marius Mihasan, Stephen T Lacmata, Marius Stefan, Jules R Kuiate, Victor Kuete

**Affiliations:** 1 Department of Biochemistry, Faculty of Science, University of Dschang, P.O. Box 67, Dschang, Cameroon; 2 Department of Biochemistry and Molecular Biology, Faculty of Biology, Alexandru Ioan Cuza University, Iasi, Romania

**Keywords:** Antibacterial, Gram-negative bacteria, Multi-drug resistant, Extract, Vegetable

## Abstract

**Background:**

Many edible plants are used in Cameroon since ancient time to control microbial infections. This study was designed at evaluating the antibacterial activities of the methanol extracts of ten Cameroonian vegetables against a panel of twenty nine Gram negative bacteria including multi-drug resistant (MDR) strains.

**Methods:**

The broth microdilution method was used to determine the Minimal Inhibitory Concentrations (MIC) and the Minimal Bactericidal Concentrations (MBC) of the studied extracts. When chloramphenicol was used as a reference antibiotic, the MICs were also determined in the presence of Phenylalanine-Arginine *β-*Naphtylamide (PAβN), an efflux pumps inhibitor (EPI). The phytochemical screening of the extracts was performed using standard methods.

**Results:**

All tested extracts exhibited antibacterial activities, with the MIC values varying from 128 to 1024 mg/L. The studied extracts showed large spectra of action, those from *L. sativa, S. edule*, *C. pepo* and *S. nigrum* being active on all the 29 bacterial strains tested meanwhile those from *Amaranthus hybridus, Vernonia hymenolepsis*, *Lactuca.carpensis* and *Manihot esculenta* were active on 96.55% of the strains used. The plant extracts were assessed for the presence of large classes of secondary metabolites: alkaloids, anthocyanins, anthraquinones, flavonoids, phenols, saponins, steroids, tannins and triterpenes. Each studied plant extract was found to contain compounds belonging to at least two of the above mentioned classes.

**Conclusion:**

These results confirm the traditional claims and provide promising baseline information for the potential use of the tested vegetables in the fight against bacterial infections involving MDR phenotypes.

## Background

Infectious diseases are still a major health concern, accounting for 41% of the global disease burden measured in terms of Disability-Adjusted Life Years (DALYS), close to all noninfectious diseases (43%) and far more than injuries (16%) [1]. One of the main causes of this problem is the widespread emergence of acquired bacterial resistance to antibiotics in such a way that the world is facing today, a serious threat to global public health [2] in the form of not only epidemics, but also pandemics of antibiotic resistance [3]. Several mechanisms have been accounted for, but active efflux plays an important role in this phenomenon [4]. The accumulation of different antibiotic resistance mechanisms within the same strains has led to the appearance of the so called superbugs, or multi-drug resistant bacteria [2]. Due to this problem of resistance to antibiotics, attention is now being shifted towards biologically active components isolated from plant species commonly used as herbal medicine, as they may offer a new source of antibacterial, antifungal and antiviral activities [5]. The potential antimicrobial properties of plants are related to their ability to synthesize several secondary metabolites of relatively complex structures possessing antimicrobial activities [6,7]. Among medicinal plants, vegetables associated to non or less-toxic effects have been shown to possess many medicinal properties [8,9] including antibacterial effects [3]. The present work was therefore designed to investigate the antibacterial effects of ten Cameroonian vegetables namely *Amarantus hybridus* Linn (Amarantaceae), *Vernonia hymenolepis* (H.F.) Hook., *Lactuca sativa* Linn. and *Lactuca capensis* Thumb. (Asteraceae), *Manihot esculenta* Crantz (Euphorbiaceae), *Phaseolus vulgaris Linn* (Fabaceae), *Cucurbita pepo* Linn and *Sechium edule* (Jacq) Sw. (Cucurbitaceae), *Solanum nigrum* Linn. and *Capsicum frutescens* L. (Solanaceae) against MDR bacteria expressing active efflux pumps

## Methods

### Plant material and extraction

The collected plant materials used in this study were harvested from Dschang, West Region of Cameroon in June 2010 and included the leaves of *Amarathus hybridus, Vernonia hymenolepis, Lactuca sativa, Lactuca capensis, Sechium edule, Manihot esculenta, Curcubiata pepo, Solanum nigrum,* the cloves of the *Green bean (Phaseolus vulgaris),* and the fruits of *Capsicum frutescens.* These plants were identified by Mr Victor Nana of the National Herbarium (Yaoundé-Cameroon) where all the voucher specimens were deposited with the corresponding reference number (Table 1).

**Table 1:** Plant species used in this study and their reported effects

**Plant (family); and voucher number**^**a**^	**Traditional uses**	**Parts used traditionally**	**Bioactive or potentially bioactive components**	**Bioactivities of extracts and/or compounds**
*Amarantus hybridus* Linn (Amarantaceae); 15630 HNC	intestinal bleeding, diarrhoea and excessive menstruation [5,10]	Leaves, seeds	flavonoids, steroids, terpenoids, cardiac glycosides [5] alkaloid, saponin, tannins, phenols, hydrocyanic acid and phytic acid [11,12]	antimicrobial [5,13]
*Vernonia calvoana* (H.F.) Hook	wounds [14],anticancer [15], fever, stomach ache, diarrhoea, hernia, spleen enlargement [16]	leaves	vernolepin [17,18], vernomenin [18], flavonoids (quercetin, apigenin, luteolin) [19]	cytotoxic [17], spasmolytic, anti-aggregating and de-aggregating activities, 2 antitumor activity, antimicrobial [20], insecticide [21], antifilarial [22]
(Asteraceae); 42401/HNC				
*Lactuca sativa* Linn; (Asteraceae); 25624/SRF.Cam	analgesic, conjunctivitis, tired eyes, Insomnia, sedative [23] insomnia, anxiety, neurosis, dry coughs, rheumatic pain [24] stimulate digestion, enhance appetite and relieve inflammation [25]	leaves	phenolic acids, triterpenoids, saponins, phytol [23], carotenoids [26], flavonoids including kaempherol [19] Lettucenin-A guaianolide sesquiterpenelactones conjugates, lactucin, deoxylactucin and lactucopicrin [27]	antimicrobial [28], antifungal, antibacterial [29], antitumor [30] antioxidating, analgesic, and anti-inflammatory [23] depressant [31] sedative, hypnotic, analgesic and anticonvulsant [32] hypoglycaemic [33] antioxidant l [34,35], and anxiolytic
*Lactuca capensis* Thumb (Asteraceae); 27743 HNC	antispasmodic, digestive, diuretic, hypnotic, narcotic and sedative properties. treatment of insomnia, anxiety, neuroses, hyperactivity in children, dry coughs, whooping cough, rheumatic pain, chronic join pains [36]	leaves	lactucarium, sesquiterpene lactone [37]	
*Sechium edule* (Jacq) SW (Cucurbitaceae); *42459/HNC*	urine retention, kidney diseases, arteriosclerosis, hypertension [38]	leaves	C-glycosyl and O-glycosyl, flavones in roots, leaves, stem and fruits [39], ascorbic acid,gibberellins, flavonoids and saponins [38]	diuretic [9], free radical-scavenger and antioxydant [40],antibacterial [41], antihypertensive [42] hepatoprotective activity of ethanolic extract and its different [43]
*Manihot esculenta* Crantz	hypertension, headache and pain, irritable bowel syndrome. fever, headache, aches and pains [44]	leaves	3-rutinosides of kaempferol and quercetin; the cyanogenic glycosides, lotaustralin and linamarin, from the fresh leaves of cassava [45]	anthelmintic activity of crude extracts antibacterial [46]
(Euphorbiaceae); 57650/HNC		leaves		
*Phaseolus vulgaris Linn* (Fabaceae)*;* 42587/HNC	osteoporosis prevention, diuretic, eczema, antiglycemic [47]	cloves	ascorbic acid, phenol, alkaloids, sterols, saponins (aqueous extract), carotenoids like lutein, β-carotene, violanthin and neoxanthin, flavonoids [48] including quercetin, kaemferol, catechins, epicatechins and procyanidins	antioxydant [48], antibacterial [49]
*Cucurbita pepo* Linn (cucurbitaceae); 15630 HNC	intestinal infections and kidney problems (seeds), minor injuries (flowers), anthelmintic, hypertension, erysipelas, enteritis, dyspepsia, stomach disorders, liver disorders like jaundice [50]	leaves	saponin, tannin, quinone, coumarins, flavonoids, sterol, terpenes, [51] lignin, alkaloids, protein and sugar Curbicin [52] anthocyanin, phenols like syringic acid [52], phytin, lecithin, cucurbitane and hexanocucurbitane L-2-O-β-glucopyranoside, Curbicin [52], flavonoids, Vitamins B, C, and E, β-sitostérol	antihypertensive, anti-oxidative activities,Arthritis, reduce the symptoms of BPH [52,53]. High Cholesterol, anti-parasitic activity in vi-vitro [54], alleviates the detrimental effects associated with protein malnutrition [55], antiparasitic[56], nephron and hepato-protective, vermifuge, inhibitor of prostaglandin biosynthesis [57], antiparasitic, protects gastric mucosal [50]
*Solanum nigrum* Linn (Solanaceae); 43000 HNC	pneumonia aching teeth, stomache ache, tonsilitis, tonic,wing worms [14], pain, inflammation and fever. tumor, antioxydant, anti-inflammatory, hepaprotective, diuretic, antipyretic [58]	leaves	kaempferol [19,59] terpenoids and condensed tannin [60], quercetin, flavonoids [19], polysaccharides, polyphenolic compounds including galic acid, catechin, cafeic acid, rutin and naringenin [58]	anti-inflammatory, antioxidant, anthelmintic activity [60] antinociceptive, antipyretic, antitumor, antiulcerogenic, cancer chemopreventive, hepatoprotective, and immunomodulatory effects [61] Mosquito larvicidal [62], antibacterial [63]
*Capsicum frutescens* L. (Solanaceae); 10737/SRFcam	wound, male virility [16], insecticide [64], rheumatism, laxative [65] relieve muscle, joint, and toothache pain, to treat cough, asthma, and sore throat, as a stimulant, treat stomach ache, seasickness, and flatulence anciently	fruits	alkaloids, flavonoids, polyphenols [66,67] and sterols [67] Capsaicin, and dihydrocapsaicin, sterols and polyterpenes, polyphenols, flavonoids, alkaloids, vitamin B2 [65], ortho- hydroxyl- N- benzyl- 16- Methyl-11,14-diene-octadecamide and 9, 12-diene-octadecanoic acid [68], carotenoids, flavonoids and saponins [68,69]	antibacterial [67], antioxydant [67], insecticidal [69]

^a^(HNC): Cameroon National Herbarium; (SRFC): Société des Réserves Forestières du Cameroun.

Air dried and powdered sample (1 kg) of each plant was extracted with methanol (MeOH) for 48 h at room temperature (25°C), using Whatman Grade No.1 filter paper and concentrated under reduced pressure, then dried to give the crude extracts. All extracts were stored at 4°C until further use.

### Preliminary phytochemical investigations

The major secondary metabolites classes such as alkaloids, anthocyanins, anthraquinones, flavonoids, phenols, saponins, tannins, sterols and triterpenes were screened according to the common phytochemical methods previously described by Harbone, 1973 [70].

### Bacterial strains and culture media

The studied bacteria included both reference (from the American Type Culture Collection) and clinical strains of *Providencia stuartii, Pseudomonas aeruginosa, K. pneumoniae, Escherichia coli, Enterobacter aerogenes* and *Enterobacter cloacae* (See Additional file 1: Table S1 for their features). These clinical strains were obtained from the laboratory “*Transporteurs Membranaires, Chimiorésistance et Drug Design, UMR-MD1, IFR 88, UFRs de Médecine et de Pharmacie, Marseille, France”.* All strains were maintained in Nutrient Broth at 4°C and activated on Mueller Hinton Agar plates 24 h prior to any antimicrobial test. Mueller Hinton Broth (MHB) was used for all antibacterial assays.

### Bacterial susceptibility testing

The MICs were determined using the rapid INT colorimetric assay [71,72]. Briefly, test samples were first emulsified in DMSO/MHB (50:50 V/V). The solution obtained was then added to MHB, and serially diluted two fold (in a 96- wells microplate). One hundred microlitres (100 μl) of inoculum (1.5 × 10^6^ CFU/ml) prepared in MHB was then added. The plate was covered with a sterile plate sealer, then agitated to mix the contents of the wells using a shaker and incubated at 37°C for 18 h. The final concentration of DMSO was 2.5% and did not affected the microbial growth. Wells containing MHB, 100 μl of inoculum and DMSO at a final concentration of 2.5% served as negative control. The MICs of samples were detected after 18 h incubation at 37°C, following addition of 40 μl of a 0.2 mg/ml INT solution and incubation at 37°C for 30 minutes. Viable bacteria reduce this yellow dye to pink. MIC was defined as the lowest sample concentration that exhibited complete inhibition of microbial growth and then prevented this change [73]. The MBC was determined by adding 50 μL of the suspensions from the wells, which did not show any growth after incubation during MIC assays, to 150 μL of fresh broth. These suspensions were re-incubated at 37°C for 48 hours. The MBC was determined as the lowest concentration of extract which completely inhibited the growth of bacteria [74].

Chloramphenicol, used as reference antibiotic, was tested also in the presence of the PAβN, at 30 mg/L final concentration to confirm the resistance of bacterial strains.

## Results

### Chemical composition of the vegetable extracts

The results of the qualitative analysis showed that each of the studied plant extract contains at least two classes of secondary metabolites such as alkaloids, anthocyanins, anthraquinones, flavonoids, phenols, saponins, steroids, tannins and triterpenes (Table 2). Only the extract from *A. hybridus* contains anthocyanins, while triterpenes were found both in this extract as well as that of *C. frutescens*. The extract from *C. frutescens* as well as those from *S. edule* and *M. esculenta* contained the highest number of classes of the studied secondary metabolites (five). Alkaloids and phenols were present in all vegetable extracts except that of *A. hybridus*.

**Table 2:** Extraction yields and phytochemical composition of the plant extracts

**Scientific names**	**Part used**	**Yield (%)**	**alkaloids**	**phenols**	**tannins**	**terpènes**	**stéroids**	**flavonoids**	**anthraquinones**	**anthocyanins**	**saponins**
Amarantus hybridus	leaves	7.9	-	-	-	+	-	+	-	+	-
*Vernonia hymenolepis*	leaves	9.40	+	+	-	-	-	+	-	-	-
*Lactuca sativa*	leaves	7.14	+	+	-	-	-	+	-	-	-
*Lactuca capensis*	leaves	7.14	+	+	+	-	+	-	-	-	-
*Sechium edule*	leaves	3.76	+	+	-	+	+	+	-	-	+
*Manihot esculinta*	leaves	07.46	+	+	+	+	+	+	+	-	+
*Phaseolus vulgaris*	cloves	17.81	+	+	-	-	+	+	-	-	-
*Cucurbita pepo*	leaves	12.68	+	+	-	-	+	+	-	-	-
*solanum nigrum*	leaves	11.84	+	+	-	-	+	+	-	-	-
*Capsicum frutescens*	fruits	16.24	+	+	-	+	-	+	+	-	-

(+): Present; (−): Absent; *The yield was calculated as the ratio of the mass of the obtained methanol extract/mass of the plant powder.

### Antibacterial activity of the vegetable extracts

The data summarized in Table 3 show the antibacterial activities of the tested extracts on a panel of twenty-nine Gram-negative bacteria. All extracts were active on at least twelve bacterial strains with MIC ≤ 1024 μg/ml. The extract of *C. frutescens* showed inhibitory activities against 16 (55.17%) of the 29 tested bacteria whilst that of *P. vulgaris* inhibited the growth of 12/29 (41.38%) pathogens (narrowest spectrum). None of these two extracts showed any antibacterial activity against *Pseudomonas* species, but were active against at least one bacterial strain of other studied genus. Extracts from *L. sativa, S. edule*, *C. pepo* and *S. nigrum* displayed the largest spectra of activity, their inhibitory effects being observed on all the 29 Gam-negative bacteria (100% of activity). The extracts from *A. hybridus*, *V. hymenolepis, L. sativa*, *L. carpensis* and *M. esculenta* also exhibited large spectrum of activity as they were active on 28/29 tested bacteria. The top eight active extracts, with large spectra of activity, showed MIC values generally ranging from 128 to 512 μg/ml. These MIC values were in some of the cases better than those of choramplenicol (Table 3). This was the case with the extract from *V. hymenolepis* (MIC of 128 μg/ml) against *E. aerogenes* EA27. The extracts from *A. hybridus, S. edule* and *C. pepo* as well as those from *L. capensis* and *M. esculenta* were more active than chloramphenicol on at least one of the tested MDR bacteria. The activity of chloramphenicol increased in the presence of PAβN in the majority of the tested bacteria (Table 3). The best activity was obtained with the extract from *A. hybridus* with the lowest MIC value of 128 μg/ml observed against 7/29 (25%) tested bacteria. The extracts from *P. vulgaris* and *C. frutescens* did not show any MBC value at up to 1024 μg/ml. Concering the eight other vegetable extracts, the MBC results showed values equal to or below 1024 μg/ml in many cases. The extract from *C. pepo* leaves showed the best MBC spectrum with the values below to 1024 μg/ml recorded on 58,62% (17/29) of the studied microorganisms, followed by those from *M. esculenta* leaves on 51,72% (15/29), *A. hybridus*, *V. hymenolepis* and *L. capensis extracts* on 44.83% (13/29) and *L. sativa* on 31.03% (9/29) (Table 4).

**Table 3:** **Susceptibility of bacteria to plant extracts - MICs of methanol extracts *vs *chloramphenicol**

**Bacteria strains**	**MIC (μg/ml) of the plant extracts**										
	***A. hybridus***	***V. hymenolepis***	***L. sativa***	***L. capensis***	***S. edule***	***M. esculenta***	***P. vulgaris***	***C. pepo***	***S. nigrum***	***C. frutescens***	**Chloramphenicol**^**1**^
	***E. coli***											
**ATCC8739**	256	1024	512	512	256	256	1024	512	512	512	4	
**ATCC10536**	128	256	128	256	128	256	-	256	128	-	4	
**W3110**	256	512	256	256	256	512	-	128	256	-	8 (< 2)	
**MC4100**	512	1024	512	1024	256	512	1024	256	512	1024	16 (< 2)	
**AG100A**	256	512	512	512	256	512	-	512	512	1024	< 2 (< 2)	
**AG100Atet**	256	512	512	512	256	512	-	512	512	1024	64 (< 2)	
**AG102**	1024	128	1024	512	512	128	-	-	256	512	64 (< 2)	
**AG100**	128	1024	128	512	512	512	-	256	128	-	8 (< 2)	
***E. aerogenes***												
**ATCC13048**	128	1024	256	256	256	256	1024	256	256	-	8	
**EA294**	512	512	512	512	512	1024	-	512	512	1024	16	
**CM64**	128	128	256	256	128	256	1024	512	256	512	256 (8)	
**EA3**	256	256	128	128	256	128	1024	128	128	-	256 (128)	
**EA298**	256	512	256	256	256	256	1024	128	256	1024	64 (< 2)	
**EA27**	512	128	256	-	512	512	-	512	256	512	≥ 256 (< 2)	
**EA289**	-	512	1024	256	512	256	1024	128	1024	256	≥ 256 (64)	
***K. pneumoniae***												
**ATCC11296**	256	512	256	512	512	512	512	256	256	-	8	
**KP55**	256	512	256	512	256	512	1024	256	256	256	32 (4)	
**KP63**	256	256	256	256	256	256	-	512	256	512	64 (< 2)	
**K2**	512	-	512	512	1024	512	-	1024	512	1024	32 (< 2)	
**K24**	512	1024	512	512	512	-	1024	512	512	1024	16 (< 2)	
***P. aeruginosa***												
**PA01**	256	512	512	256	256	512	-	256	512	-	16	
**PA124**	512	1024	512	512	512	512	-	512	512	-	32 (< 2)	
***P. stuartii***												
**ATCC29916**	128	128	256	1024	128	1024	-	1024	256	-	16	
**NAE16**	128	512	256	256	256	256	1024	512	256	-	64 (8)	
**PS2636**	512	512	256	256	256	256	-	256	256	512	32	
**PS299645**	512	1024	1024	512	512	512	-	512	1024	-	32 (< 2)	
***E. cloacae***												
**BM47**	128	256	512	1024	256	1024	-	128	512	-	≥ 256 (< 2)	
**ECCI69**	256	512	512	256	256	128	-	256	512	-	≥ 256 (16)	
**BM67**	256	512	512	256	256	512	1024	128	512	1024	128 (32)	

The results are shown as average values from three separate experiments.

(−) MIC > 1024 μg/ml.

^1^ - chloramphenicol was used as a reference antibiotic. MIC was measured in absence and presence of PAßN (in brackets).

**Table 4:** **Susceptibility of bacteria to plant extracts - MBCs (μg/ml) of methanol extracts *vs *chloramphenicol**

**Bacteria strains**	***A. hybridus***	***V. hymenolepis***	***L. sativa***	***L. capensis***	***S. edule***	***M. esculenta***	***Green bean (P. vulgaris)***	***C. pepo***	***S. nigrum***	***C. frutescens***	**Chloramphenicol**^**1**^
***E. coli***											
**ATCC8739**	-	-	-	-	1024	1024	-	512	-	-	64
**ATCC10536**	1024	-	-	-	-	-	-	1024	-	-	128
**W3110**	1024	512	256	-	512	-	-	512	-	-	-
**MC4100**	1024	-	-	-	-	-	-	-	-	-	-
**AG100A**	-	1024	512	-	-	512	-	-	-	-	-
**AG100Atet**	-	1024	512	-	-	-	-	-	-	-	-
**AG102**	-	-	512	1024	-	-	-	-	-	-	-
**AG100**	256	1024	-	1024	-	512	-	1024	-	-	-
***E. aerogenes***	-	-	-		-				512		
**ATCC13048**				1024		1024	-	1024		-	128
**EA294**	-	-	-	-	-	1024	-	-	-	-	32
**CM64**	512	-	-	512	512	512	-	-	-	-	-
**EA3**	1024	512	1024	1024	-	512	-	1024	1024	-	-
**EA298**	512	1024	1024	-	1024	256	-	256	512	-	-
**EA27**	-	-	-	-	-	512	-	-	-	-	-
**EA289**	-	1024	-	512	1024	512	-	-	-	-	-
***K. pneumoniae***-											
**ATCC11296**	-	-	-	1024	-	-	-	256	1024	-	64
**KP55**	1024	-	-	-	1024	1024	-	1024	512	-	128
**KP63**	512	512	-	-	-	-	-	512	1024	-	-
**K2**	1024	-	-	1024	-	-	-	-	-	-	256
**K24**	-	-	-	-	1024	-	-	512	-	-	512
***P. aeruginosa***											
**PA01**	-	-	-	-	-	-	-	-	-	-	256
**PA124**	-	1024	-	1024	-	-	-	1024	512	-	-
***P. stuartii***											
**ATCC29916**	-	256	-	1024	1024	1024	-	1024	512	-	128
**NAE16**	-	-	512	-	1024	-	-	1024	-	-	256
**PS2636**	512		1024	1024	-	1024	-	512	-	-	-
**PS299645**	-	-	-	-	-	-	-	-	-	-	-
***E. cloacae***											
**BM47**	-	1024	512	-	-	-	-	-	1024	-	-
**ECCI69**	1024	512	-	1024	512	1024	-	1024	512	-	-
**BM67**	512	1024	-	1024	1024	1024	-	1024	-	-	-

The results are shown as average values from three separate experiments.

(−) MBC > 1024 μg/ml.

^1^ - chloramphenicol was used as a reference antibiotic.

Table 4 also shows that *M. esculenta* exhibited MBC values against all the strains of *E. aerogenes* and that, in general, the extracts showed values which were not 4-fold greater than the corresponding MICs.

## Discussion

In plants, secondary metabolites attract beneficial and repel harmful organisms, serve as phytoprotectants and respond to environmental changes. In animals, such compounds have many beneficial effects including antibacterial and antiviral properties [75,76]. The classes of secondary metabolites detected in the tested vegetables can somehow provide a prelimanry explanation on their activities [77]. In general, the phytochemical contents (Table 2) were in accordance with the previous reports for some of the vegetables where data were available [11,12,23,38]. It should however be mentioned that the detection of the bioactive phytochemical classes in a plant is not a guarantee for any biological property, as this will depend on the types of compounds, as well as their concentrations and possible interaction with other constituents.

*Solanum nigrum* has been shown to possess various activities such as antitumorigenic, antioxidant, anti-inflammatory, hepatoprotective diuretic and antipyretic [63]. Though the exact mechanism of action remains to be elucidated in many cases, few are known about its antibacterial properties. In fact, it has been shown that seeds of *S. nigrum* possess good antimicrobial activity against *E. coli* on solid medium [63]. We report herein for the first time the antibacterial activity of leaves methanol extract of this plant against a panel of MDR Gram-negative bacterial strains with MIC values varying from 128 to 1024 μg/ml (Table 3). *Solanum nigrum* possesses various compounds that are responsible for diverse activities. Among these compounds, solanine (found in all parts of the plant [58]),is its major defence product [58].

Many reports have also been published about the biological properties of *C. pepo*, but these reports are based on the components of the fruits and the seed’s oil [54,55,57,78]. To the best of our knowledge, were herein report for the first time its activities against MDR bacteria.

The results of the phytochemical test on *P. vulgaris* are in accordance with some other reports [48,79]. *Phaseolus vulgaris* was found to inhibit also the growth of Gram-positive bacteria *B.subtilis*[49]. Amarowicz et al. [80] showed that the acetone extract of *P. vulgaris* contains tannins with good antimicrobial properties against *Listeria monocytogenes*. Therefore, the low antibacterial effects of this plant as obtained herein (generally MIC values at 1024 μg/ml) (Table 3) could be due to the multi-drug resistance ability of the studied bacteria.

The antibacterial effects of the extract from *C. frutescens* against *Staphylococcus aureus* as well as *K. pneumoniae and P. aeruginosa* have been reported [67]. The ethanol extract of this plant was also active against MDR strains of *S. aureus*[81]. The present study therefore provides additional information on the antibacterial potential of this plant on MDR Gram-negative bacteria with MICs ranging from 256 to 024 μg/ml.

The antibacterial properties of *S. edule* have already been proved against bacteria of clinical relevance by Ordonez et al. [41] which showed that both fluid extract and tincture of fruits have “very good*”* antimicrobial activities against MDR staphylococci and enterococci [41]. Herein, the antimicrobial activity of the leaves extract {known to possess high level of secondary metabolites and mostly flavonoids [39]} observed against all the studied bacterial strains (Table 3) is being reported for the first time.

The chloroform extract of *M. esculenta* possess antibacterial activities against *Listeria monocytogenes, Vibrio cholerae, Shigella flexneri* and *Salmonella typhi* whilst ethanol extract was found active against *P. aeruginosa*, *Corynebacterium diphtheriae* and *V. cholera*[46]. This report provides additional data on antibacterial activity of *M. esculenta* against MDR strains of *P. aeruginosa, E. coli, E. cloacae, K. pneumoniae, P stuartii* and *E. aeorogenes*. The activity of *Amaranthus hybridus* was reported against *E. coli, S. typhi, K. pneumoniae* and *P. aeruginosa* with MICs ranged between 200 and 755 mg/ml [5]. The ethyl acetate extract exhibited activity against *S. aureus* and *B. subtilis* whilst the ethanol extract was found effective against *E.coli*[13].

The high MIC values observed with chloramphenicol can be explained only if we take into account the non-specific resistance mechanism: active efflux of the toxic compound by pumps belonging to the small multidrug resistance (SMR) proteins family [4]. The fact that the efflux pump inhibitor (PAβN) enhances the chloramphenicol antibacterial properties is a clear indication that the tested strains express an active efflux system and that this system is responsible for resistance of the tested bacteria to chloramphenicol. The wide substrate specificity of these pumps, as well as their widespread among bacterial species make us believe that these efflux pumps are also responsible for the extrusion of various active compounds from the plant extract out of bacteria cells, therefore preventing their inhibitory effects. Therefore, the activities of the vegetable as observed herein against MDR strains (with MIC comprised between 128 and 1024 μg/mL) could be considered important, especially when considering the fact that we are dealing with edible plants. Apart for the extracts of *P. vulgaris* and *C. frutescens* which did not show any MBC below 1024 μg/ml, other values further confirmed the bactericidal effect of the 8 remaining extracts as they were generally less than 4-fold greater than corresponding MIC values [82,83].

## Conclusions

The overall results of the present investigation confirmed the traditional uses of the studied vegetables in the treatment of bacterial infections. This study also provide baseline information for the possible use of the methanol extracts of the tested plant samples in the control of infectious diseases involving Gram-negative MDR bacteria. The arising question is of course which are the active compounds responsible for these effects. Our research group is currently focusing on the characterization of these plants extracts in terms of chemical composition and synergistic effects.

## Competing interest

The authors declare that they have no competing interest.

## Authors’ contributions

JAKN*,* MM, STL and MS carried out the study; VK designed the experiments. JAKN, MM and VK wrote the manuscript; VK and JRK supervised the work; VK provided the bacterial strains; all authors read and approved the final manuscript.

## Pre-publication history

The pre-publication history for this paper can be accessed here:

http://www.biomedcentral.com/1472-6882/13/26/prepub

## Supplementary Material

Additional file 1: Table S1 Bacterial strains and features. Click here for file
