# Supplementary material for: Antibacterial activities of the methanol extracts of ten Cameroonian vegetables against Gram-negative multidrug-resistant bacteria
Source: BMC Complement Altern Med. 2013 Jan 31;13:26. doi: 10.1186/1472-6882-13-26 (PMC3598735; doi:10.1186/1472-6882-13-26)
Supplement: Additional file 1: Table S1 — Bacterial strains and features. [file 1472-6882-13-26-S1.doc]

**Antibacterial activities of the methanol extracts of ten Cameroonian vegetables against Gram-negative multidrug-resistant bacteria**

Jaurès A.K. Noumedem^a,b^, Marius Mihasan^b^, Jules R. Kuiate^a^ , Stephen T. Lacmata^a^, Marius Ştefan^b^, Dumitru Cojocaru^b^ and Victor Kuete^a*^

*^a^Department of Biochemistry, Faculty of Science, University of Dschang, Dschang-Cameroon*

*^b^Department of Biochemistry and Molecular Biology, Faculty of Biology, University Alexandru Ioan Cuza, Iasi-Romania*

****Corresponding author:***

*Tel : 77 35 59 27 ; Fax: (237) 22 22 60 18. P.O. Box 67 Dschang, Cameroon; E-mail: kuetevictor@yahoo.fr (Dr. Victor Kuete)*

**[See Main Manuscript]**

**Table S1. Bacterial strains and features**

| **Strains** | **Features** | **References** |
| --- | --- | --- |
| ***Escherichia coli*** |  |  |
| ATCC8739 and ATCC10536 | Reference strains |  |
| AG100 | Wild-type *E. coli* K-12 | [1] |
| AG100A | AG100 *ΔacrAB*::KAN^R^ | [1, 2] |
| AG100A_TET_ | Δ*acrAB* mutant AG100, owing *acrF* gene markedly over-expressed; TET^R^ | [1] |
| AG102 | Δ*acrAB* mutant AG100 | [3] |
| MC4100 | Wild type *E. coli* |  |
| W3110 | Wild type *E. coli* | [4, 5] |
| ***Enterobacter aerogenes*** |  |  |
| ATCC13048 | Reference strains |  |
| EA-CM64 | CHL^R^ resistant variant obtained from ATCC13048 over-expressing the AcrAB pump | [6] |
| EA3 | Clinical MDR isolate; CHL^R^, NOR^R^, OFX^R^, SPX^R^, MOX^R^, CFT^R^, ATM^R^, FEP^R^ | [7] |
| EA27 | Clinical MDR isolate exhibiting energy-dependent norfloxacin and chloramphenicol efflux with KAN^R^ AMP^R^ NAL^R^ STR^R^ TET^R^ | [7] |
| EA289 | KAN sensitive derivative of EA27 | [8] |
| EA298 | EA 289 *tolC::*KAN^R^ | [8] |
| EA294 | EA 289 *ΔacrAB*: *::*KAN^R^ | [8] |
| ***Enterobacter cloacae*** |  |  |
| ECCI69 | Clinical isolates | Laboratory collection of UNR-MD1, University of Marseille, France |
| BM47 | Clinical isolates | Laboratory collection of UNR-MD1, University of Marseille, France |
| BM67 | Clinical isolates | Laboratory collection of UNR-MD1, University of Marseille, France |
| ***Klebsiella pneumoniae*** |  |  |
| ATCC12296 | Reference strains |  |
| KP55 | Clinical MDR isolate, TET^R^ , AMP^R^, ATM^R^, CEF^R^ | [9] |
| KP63 | Clinical MDR isolate, TET^R^, CHL^R^, AMP^R^, ATM^R^ | [9] |
| K24 | AcrAB-TolC | Laboratory collection of UNR-MD1, University of Marseille, France |
| K2 | AcrAB-TolC | Laboratory collection of UNR-MD1, University of Marseille, France |
| ***Providencia stuartii*** |  | **[10]** |
| NEA16 | Clinical MDR isolate, AcrAB-TolC |
| ATCC29914 | Clinical MDR isolate, AcrAB-TolC |
| PS2636 | Clinical MDR isolate, AcrAB-TolC |
| PS299645 | Clinical MDR isolate, AcrAB-TolC |
| ***Pseudemonas aeruginosa*** |  |  |
| PA 01 | Reference strains |  |
| PA 124 | MDR clinical isolate | [11] |

^a^AMP, ATM^R^, CEF^R^, CFT^R^, CHL^R^, FEP^R^, KAN^R^, MOX^R^, STR^R^, TET^R^. Resistance to ampicillin, aztreonam, cephalothin, cefadroxil, chloramphenicol, cefepime, kanamycin, moxalactam, streptomycin, and tetracycline; MDR : Multidrug resistant.

1. Viveiros M, Jesus A, Brito M, Leandro C, Martins M, Ordway D, Molnar M, Molnar J, Amaral L: **Inducement and reversal of tetracycline resistance in *Escherichia coli* K-12 and expression of proton gradient-dependent multidrug efflux pump genes**. *Antimicrobial Agents Chemotherapy* 2005, **^49^**^(8):3578-3582^.

2. Okusu H, Ma D, Nikaido H: **AcrAB efflux pump plays a major role in the antibiotic resistance phenotype of *Escherichia coli* multiple-antibiotic- resistance Mar mutants.** . *Journal of Bacteriology* 1996, **^178^**^(1):306-308^.

3. Elkins CA, Mullis LB: **Substrate competition studies using whole-cell with the major tripartite multidrug efflux pumps of *Escherichia coli***. *Antimicrobial Agents Chemotherapy* 2007, **^1^**^(3):923-929^.

4. Baglioni P, Bini L, Liberatori S, Pallini V, Marri L: **Proteome analysis of *Escherichia coli* W3110 expressing an heterologous sigma factor**. *Proteomics* 2003, **^3^**^(6):1060-1065^.

5. Sar C, Mwenya B, Santoso B, Takaura K, Morikawa R, Isogai N, Asakura Y, Toride Y, Takahashi JA: **Effect of *Escherichia coli* wild type or its derivative with high nitrite reductase activity on in vitro ruminal methanogenesis and nitrate/nitrite reduction**. *Journal of Animal Science* 2005, **^83^**^:644-652^.

6. Ghisalberti D, Masi M, Pagès J-M, Chevalier J: **Chloramphenicol and expression of multidrug efflux pump in Enterobacter aerogenes**. *Biochemical and Biophysical Research Communications* 2005, **^328^**^(4):1113-1118^.

7. Malléa M, Mahamoud A, Chevalier J, Alibert-Franco S, Brouant P, Barbe J, Pagès JM: **Alkylaminoquinolines inhibit the bacterial antibiotic efflux pump in multidrug-resistant clinical isolates**. *Biochemistry Journal* 2003, **^376^**^:801-805^.

8. Pradel E, Pagès J-M: **The AcrAB-TolC efflux pump contributes to multidrug resistance in the nosocomial pathogen *Enterobacter aerogenes*** *Antimicrobial Agents Chemotherapy* 2002, **^46^**^(2640-2643)^.

9. Chevalier J, Pagès J-M, Eyraud A, Malléa M: **Membrane Permeability Modifications Are Involved in Antibiotic Resistance in *Klebsiella pneumoniae***. *Biochemical and Biophysical Research Communications* 2000, **^274^**^(2):496-499^.

10. Tran Q-T, Mahendran KR, Hajjar E, Ceccarelli M, Davin-Regli A, Winterhalter M, Weingart H, Pagès J-M: **Implication of Porins in β-Lactam Resistance of *Providencia stuartii***. *The Journal of Biological chemistry* 2010, **^285^**^(42):32273–32281^.

11. Lorenzi V, Muselli A, Bernardini AF, Berti L, Pagès JM, Amaral L, Bolla JM: **Geraniol restores antibiotic activities against multidrug-resistant isolate from Gram-negative species.** . *Antimicrob Agents Chemotherapy* 2009, **^53^**^: 2209-2211^.
